# Light Guide Layer Thickness Optimization for Enhancement of the Light Extraction Efficiency of Ultraviolet Light–Emitting Diodes

**DOI:** 10.1186/s11671-021-03563-6

**Published:** 2021-06-13

**Authors:** Zhi Ting Ye, Yuan-Heng Cheng, Li-Wei Hung, Kung-Hsieh Hsu, Yu Chang Hu

**Affiliations:** 1 Department of Mechanical Engineering, Advanced Institute of Manufacturing With High-Tech Innovations, National Chung Cheng University, 168, University Rd., Min-Hsiung, Chia-Yi, 62102 Taiwan; 2 Department of Process Development Division, EPILEDS TECHNOLOGIES, No. 7, Kanxi Rd., Xinshi Dist., Tainan City, 744092 Taiwan; 3 Department of R&D Division, Harvatek Corporation, No. 18, Ln. 522, Sec. 5, Zhonghua Rd., Xiangshan Dist., Hsinchu City, 300066 Taiwan

**Keywords:** Deep-ultraviolet light-emitting diodes, Light extraction efficiency, Light guide layer, First-order optical design

## Abstract

Consider material machinability and lattice mismatch sapphire as substrates for the ultraviolet-C light-emitting diodes (UV-C LEDs) are commonly used, but their high refractive index can result in the total internal reflection (TIR) of light whereby some light is absorbed, therefore caused reducing light extraction efficiency (LEE). In this study, we propose a method to optimize the thickness of a sapphire substrate light guide layer through first-order optical design which used the optical simulation software Ansys SPEOS to simulate and evaluate the light extraction efficiency. AlGaN UV-C LEDs wafers with a light guide layer thickness of 150–700 μm were used. The simulation proceeded under a center wavelength of 275 nm to determine the optimal thickness design of the light guide layer. Finally, the experimental results demonstrated that the initial light guide layer thickness of 150 μm the reference output power of 13.53 mW, and an increased thickness of 600 um resulted in output power of 20.58 mW. The LEE can be increased by 1.52 times through light guide layer thickness optimization. We propose a method to optimize the thickness of a sapphire substrate light guide layer through first-order optical design. AlGaN UV-C LEDs wafers with a light guide layer thickness of 150–700 μm were used. Finally, the experimental results demonstrated that the LEE can be increased by 1.52 times through light guide layer thickness optimization.

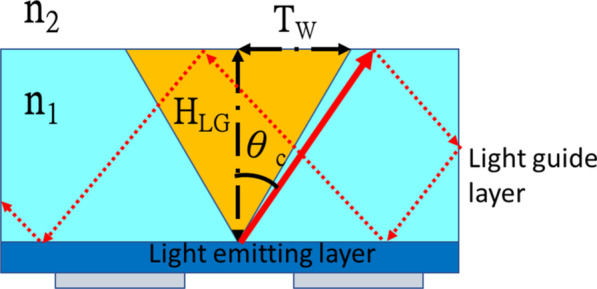

## Introduction

The COVID-19 pandemic has led to an increase in the global mortality rate. Although traditional ultraviolet (UV)-C mercury lamps can be sterilized, their mercury content, dispersed spectral wavelength, bulkiness, and short lifetime limits their applicability. UV-C light-emitting diodes (LEDs) are environmentally friendly, mercury free, and nonpolluting. The sterilization wavelength is concentrated between 260 and 280 nm. Because the light source is small and has a long lifetime, it has gradually replaced UV-C mercury lamps as the primary sterilization light source. UV light destroys bacterial DNA or RNA structures and has been widely used to decontaminate surfaces, air, and water. The UV-C waveband between 260 and 280 nm has the greatest bactericidal effect, preventing the regeneration of microbial cells to achieve disinfection and sterilization [1–3]. Studies have documented the wide use of UV-C LEDs in medical phototherapy and in the disinfection and sterilization of water, food, and medicine for safe consumption [4–7]. Traditional mercury UV lamps are disadvantaged by their long warm-up times, short lifetime, risk of exploding, and environmental pollution; UV-C LEDs are superior in all aforementioned aspects [8–10]. The UV-C wavelength range is 100–280 nm, and the UV-C LED wavelength falls between 260 and 280 nm. Because the emission wavelength of LEDs is more concentrated, their sterilization efficiency and long-term reliability are also better than those of mercury UVlamps [11, 12]. However, the poor external quantum efficiency (EQE) and light extraction efficiency (LEE) of UV-C LEDs must be improved. The low EQE and LEE of AlGaN-based UV-C LEDs are attributable to electron leakage and total internal reflection (TIR), which cause photons to be absorbed by the sapphire substrate and the materials in the p-GaN contact layer [13–15].

Approaches toward LEE improvement have involved using a nanopatterned sapphire substrate as a substrate for manufacturing UV-C LEDs. The growth of InGaN-based LED mixed patterned sapphire substrates at the microscale and nanoscale was proposed by Wen Cheng Ke et al*.*, who allowed the LED to embed nanoholes in the micropatterned sapphire substrate to improve its photoelectric characteristics [16]. PhillipManley et al. employed a nanopatterned sapphire substrate in deep UV (DUV) LEDs, verifying the effects of such a nanopatterned structure on the LEE of sapphire [17].

Shao Hua Huang et al*.* employed wet-etching of a flip-chip structure to modify a sapphire substrate and give it bevel texture, improving the LEE of a nitride LED [18]. Dong Yeong Kim proposed an n-type GaN micromirror with an Al-coated slope barrier called a sidewall emission-enhanced DUV LED to improve the LEE of transverse magnetic polarization [19].

Some scholars have proposed changing the light path to improve LEE through the design of a secondary lens. For example, Renli Liang et al*.* used nanolens arrays to enhance the LEE of DUV LEDs through lithographic and wet-etching technology. Bin Xie et al*.* proposed a freeform lens with a brightness enhancement film to enhance the overall performance of a direct-illuminated LED backlight [20, 21]. UV-C LEDs and their characteristics related to organic material absorption influence the choice of packaging materials. Nagasawa and Hirano promoted the use of p-type butyl vinyl ether with a trifluoromethyl end structure on AlGaN substrates as the encapsulated material to improve LEE [22]. Under long-term DUV irradiation, organic materials are subjected to severe molecular dissociation and destruction. To promote more efficient and reliable light extraction, a material with high resistance to UV light or inorganic materials is required. The airtightness of a package is also a key factor for evaluating packaging capability [23, 24]. To account for both high penetration and long-term reliability, quartz glass is often used as the packaging material for UV LEDs. When the cavity is hollow, high interface reflections reduce LEE; the cavity can be filled with liquid or organic glue with a low refractive index for LEE improvement. In this regard, Chieh-Yu Kang proposed a new type of DUV LED liquid packaging structure can achieve LEE improvements. Chien Chun Lu demonstrated the higher and more reliable LEE of UV-C LEDs with a quartz-based hermetic package [25, 26].

Different packaging materials such as polydimethylsiloxane (PDMS) fluid doped with SiO_2_ nanoparticles can improve the LEE of UV LEDs. Zhi Ting Ye proposed the nanoparticle-doped PDMS fluid enhanced the optical performance of AlGaN-based DUV LEDs [27]. Yang Peng employed this encapsulation material doped with fluoropolymer on an aluminum nitride substrate to enhance the LEE of a chip-on-board encapsulation structure [28]. Joosun Yun and Hideki Hirayama proposed different wafer structures in a comparative study with six different flip-chip structures, obtaining an AlGaN meta-surface for improved LEE [29].

It is also worth mentioning that photon management has been demonstrated as an efficient way to extract and harvest light and has been widely used in a variety of optoelectronic devices, including photodetectors and photoelectron chemical cells [30–33], solar cells [34, 35], and Micro-light-emitting diodes in display technology [36].

Research into the refinement of UV-C LEDs has yet to examine the effects of light guide layer thickness on LEE. When sapphire is used as the light guide layer material, the absorption rate is relatively low in the general blue wavelength band of 450 nm but relatively high in the UV-C LED 260–280 nm wavelength band, demonstrating the influence of thickness on LEE. Therefore, in this paper, an optimal value for the thickness of the light guide layer for the LEE of UV-C LEDs is proposed.

## Methods

### TIR Phenomenon in the Light Guide Layer

**Fig. 1:**
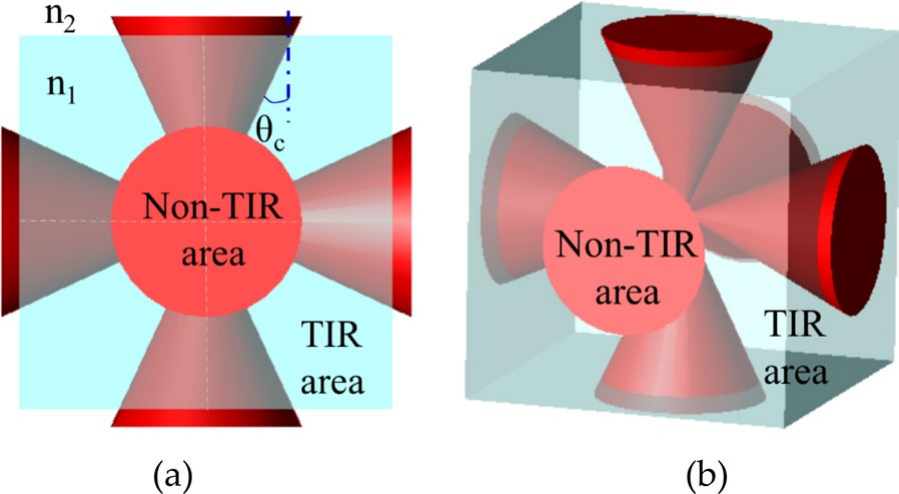
Total reflection inside the light guide layer. **a** Flat diagrammatic sketch and **b** three dimensional diagrammatic sketch

TIR is an optical phenomenon whereby the refractive index changes when light enters different media. When the incident angle is less than the critical angle, the light is divided into two parts; one part of the light is reflected and the other is refracted. Conversely, when the incident angle is greater than the critical angle, all light is internally reflected without refraction. The refractive index of the internal medium is n_1_, and the refractive index of the external medium is n_2_. The critical angle *θ*_*c*_ can be calculated using Eq. (1). When n_1_ is 1.788, the critical angle *θ*_*c*_ of the TIR is 34.136°, as illustrated in Fig. 1. The red triangle cone represents the non-total reflection area that can penetrate the light guide layer and then exit it, and the remaining cyan area is the TIR area, wherein light bounces and is absorbed by the material, reducing the LEE.

1 $${\theta }_{C}={\mathrm{sin}}^{-1}\frac{{n}_{2}}{{n}_{1}}$$

**Fig. 2:**
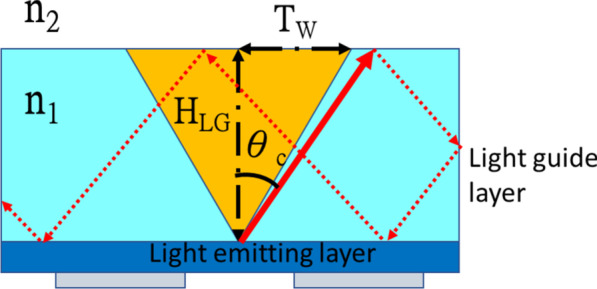
Schematic of the UV-C LED TIR phenomenon

When the length *L* and width *W* of the light guide layer are fixed, the thickness of the light guide layer H_LG_ affects the TIR area. As depicted in Fig. 2, light exits from the light-emitting layer into the light guide layer and thus, the TIR phenomenon does not occur in the orange area. If the incident angle exceeds this area, TIR occurs in cyan area of Fig. 2. The width of this area can be defined as T_W_, as expressed in Eq. (2).

2 $${T}_{W}=\mathrm{tan}({\mathrm{sin}}^{-1}\frac{{n}_{2}}{{n}_{1}})\times {H}_{LG}$$

### Simulation and Optimization of the Light Guide Layer Thickness to Enhance the LEE of UV-C LEDs

We used Solidwork 3D drawing software and Ansys SPEOS optical simulation software to construct the optical system and to simulate and optimize the effects of light guide layer thickness on LEE using first-order optical design. With Al_2_O_3_ acting as the light guide layer material, we modified the thickness to reduce absorption problems caused by TIR.

**Fig. 3:**
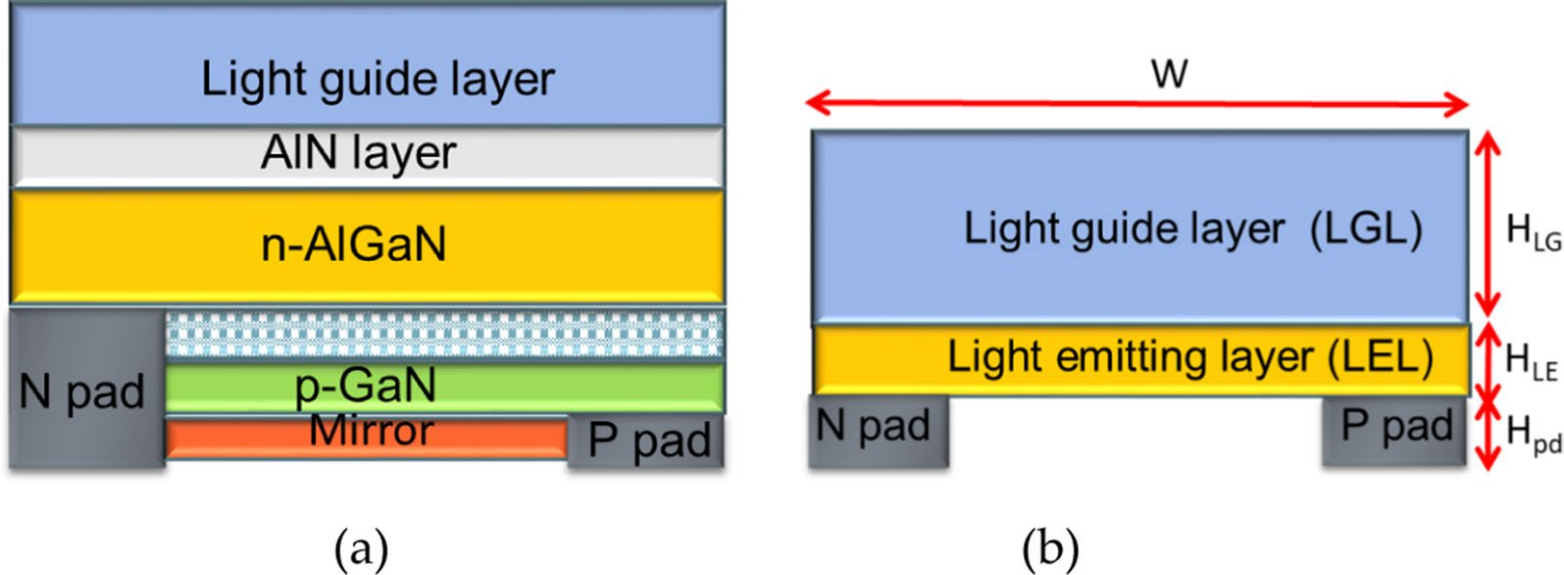
**a** Structural diagram of the UV-C LED chip, and **b** a simplified parameter diagram of UV-C LED chip simulation

The wavelength of the UV-C LED chip was 275 nm, the length *L* 1.524 mm, and the width *W* was 1.524 mm, as presented in Fig. 3.

The light guide layer was composed of Al_2_O_3_, the refractive index N_LGL_ was 1.782, and the light guide layer thickness (H_LG_) interval was 150–700 μm. The light-emitting layer (LEL) had a thickness H_LE_ of 1.5 μm, the upper surface of the layer was a light-emitting surface, the lower surface was a partially absorbing and partially reflective layer, and the UV-C LED electrode thickness H_pd_ was 1.5 μm; the material was set to partially absorb and partially reflect. Figure 3a illustrates the structure of the UV-C LED chip, and Fig. 3b is a simplified simulation diagram of the chip. The parameter settings are listed in Table 1.

**Table 1:** UV-C LED chip simulation set parameters

Item	Characteristics	Value
Input power	Radiant flux	1 W
Wavelength	Polished	275 nm
Light guide layer (LGL)	Polished	Thickness: *H*_LG_ = 150 µm–700 µm Refractive index: *N*_LGL_ = 1.782,
Light emitting layer(LEL)	Polished	Thickness: *H*_LE_ = 1.5 um Refractive index: *N*_LEL_ = 1.782
P/N electrode layer	Cr/Au/Sn	Thickness: *H*pd = 1.5 um

**Fig. 4:**
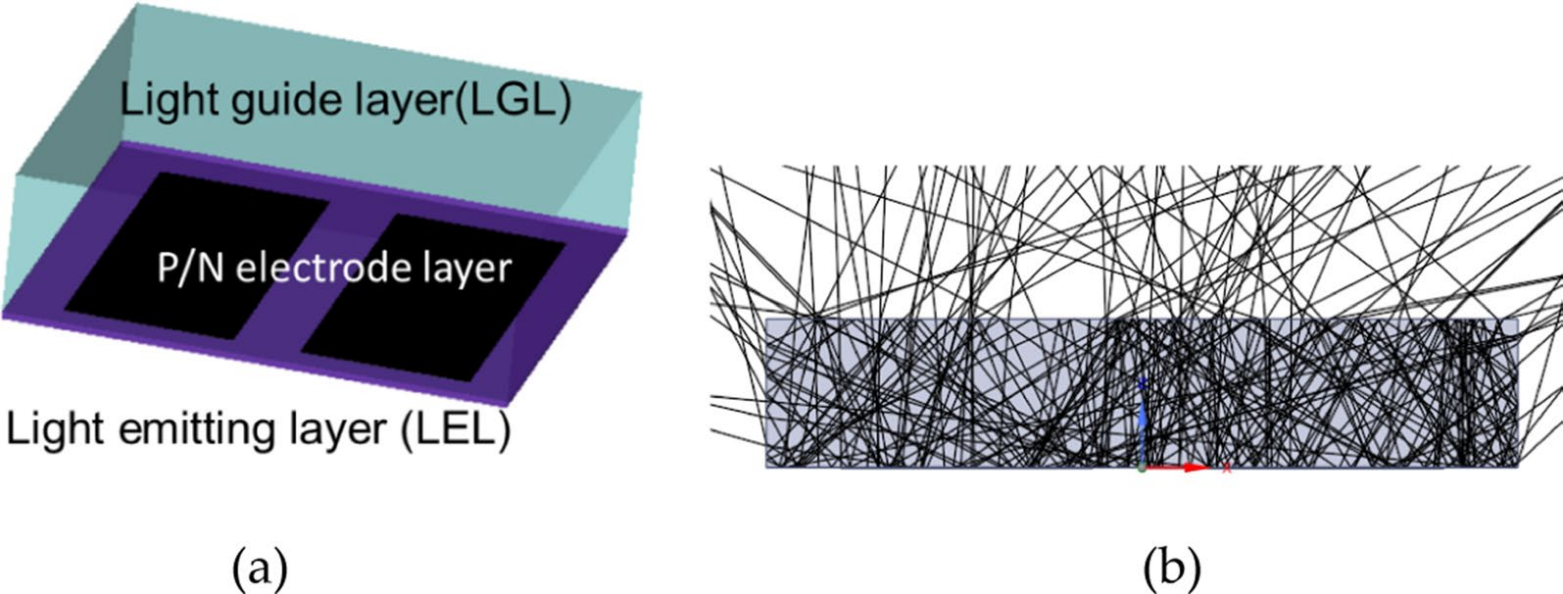
Structure of the UV-C LED; **a** three dimensional structure of the UV-C LED simulation, and **b** light trace simulation diagram

Figure 4a presents a schematic of the UV-C LED three dimensional structure, and Fig. 4b is a schematic of the light trace of the simulated light-emitting surface.

**Fig. 5:**
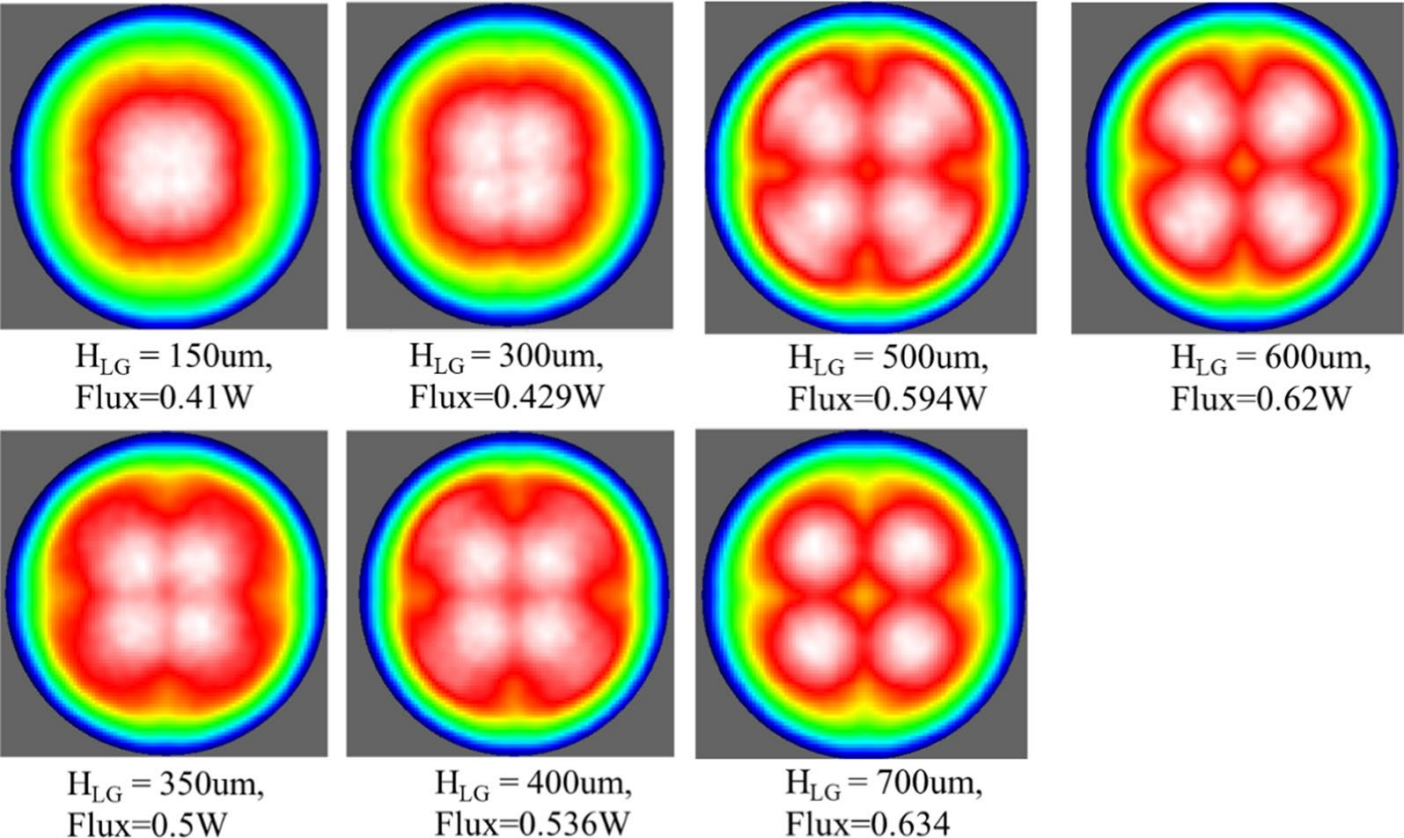
LEE diagram of the simulated UV-C LED light guide with a thickness of 150–700 μm

This study analyzed the effects of light guide thickness from 150–700 μm on LEE; the simulated input radiant flux was 1 W, and the simulation result is presented in Fig. 5. When the thickness of the light guide was 150 μm, the relative radiant flux was 0.41 W, and when the thickness of the light guide was increased, the LEE increased in turn. At a 600-μm light guide thickness, the radiant flux was 0.62 W, a 1.512-fold increase. According to the simulation results, if the thickness is further increased, the LEE is close to saturation and does not increase. When the thickness of the light guide layer was 700 μm, the efficiency was only 2.2% higher than that of the layer at 600 μm, as presented in Fig. 5.

Table 2 shows the relative radiant flux output and its magnification when the simulated radiant flux input was 1 W. The light guide layer with a thickness of 600 μm achieved the best LEE, magnification, and processing stability; however, at 700 μm, it resulted in processing and cutting difficulties and a consequent decrease in yield.

**Table 2:** LEE simulation results for a light guide layer thickness of 150–700 μm

thickness (um)	Input radiation flux (Watt)	λ (nm)	(Relative radiant flux) mW	(Radiant flux magnification)
150	1	275	0.41	1
300			0.429	1.046
350			0.5	1.219
400			0.536	1.307
500			0.594	1.448
600			0.62	1.512
700			0.634	1.546

We propose the light guide layer thickness optimization for enhancement of the LEE compared to the nano-patterned sapphire substrate method, the advantages of the method do not need to go through the etching and embossing process.

## Results and Discussion

**Fig. 6:**
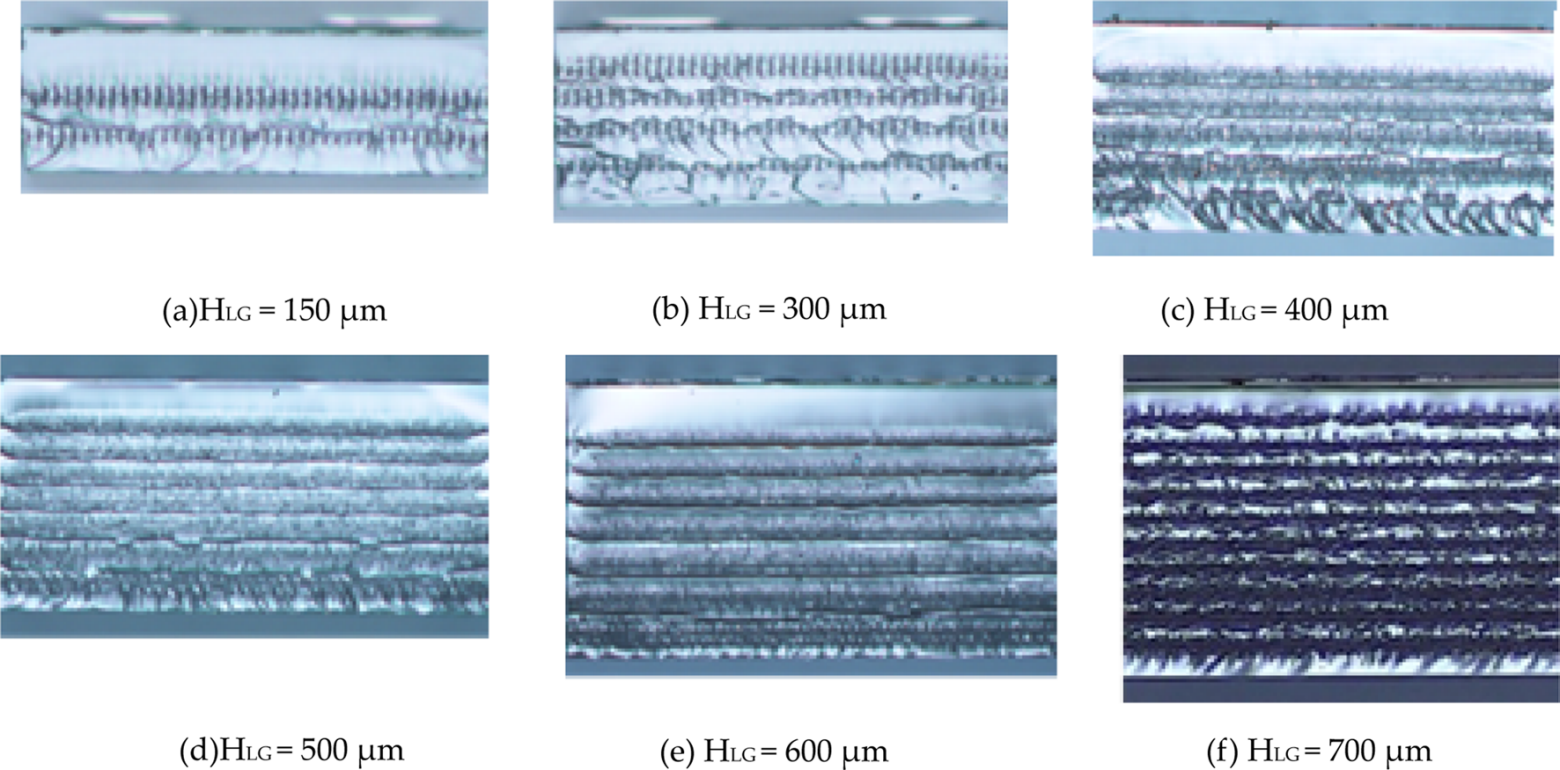
Side view of real UV-C LED samples with light guide layer thicknesses (*H*_LG_) of **a** 150, **b** 300, **c** 400, **d** 500, **e** 600, and **f** 700 μm

Figure 6 illustrates the UV-C LED prototypes with different light guide layer thicknesses (H_LG_). Figure 6a shows a H_LG_ value of 150 μm, the thickness parameter commonly used in industry settings that served as a reference measurement for this experiment. Figure 6e shows a H_LG_ of 600 μm, which is the optimal thickness for heightened LEE. In the industrial manufacturing process, increasing the thickness of the light guide layer will cause difficulty in cutting and lead to splitting problems. When the thickness of the light guide layer is 600um, it has reached the limit thickness of processing in the industry.

**Fig. 7:**
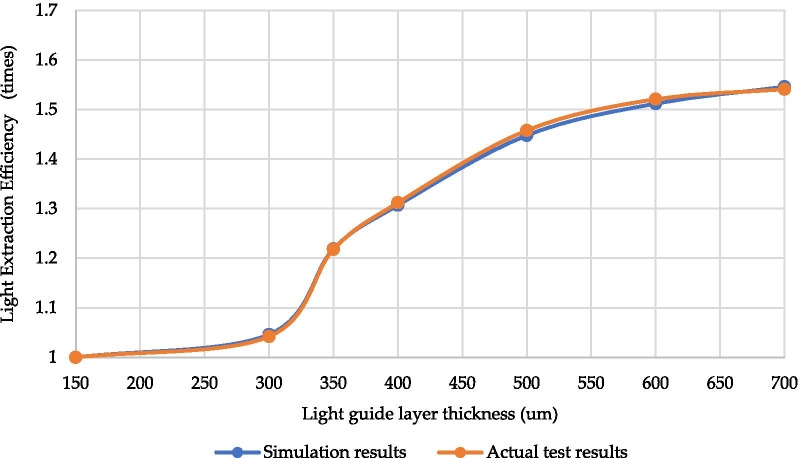
Compared simulated and measured LEE enhance times of UV-C LEDs with a light guide layer thickness of 150–700 um

Table 3 lists the relative radiant flux of the different light guide layer thicknesses (H_LG_). With H_LG_ of 600 μm, the radiant flux was 1.52 times higher than with a thickness of 150 μm. Figure 7 illustrates the UV-C LED prototype simulation and measured LEE growth trend with different light guide layer thicknesses (150–700 μm); at H_LG_ of 700 μm, the growth rate was no longer obvious and had approached saturation. The simulation results are similar to those in the actual sample test.

**Table 3:** LEE Results for light guide layer thicknesses of 150–700 μm

H_LG_ (um)	Measured of Relative radiant flux		
	λ (nm)	Relative radiant flux (mW)	Radiant flux magnification
150	275	13.53	1
300	274	14.1	1.042
350	275	16.49	1.218
400	274	17.76	1.312
500	278	19.74	1.458
600	277	20.58	1.521
700	278	20.86	1.541

Table 4 details the effects of the simulated UV-C LED on LEE under different light guide layer thicknesses; When the thickness of the light guide was 150 μm, the relative radiant flux was 13.53 mW, and when the thickness of the light guide was increased, the LEE increased in turn. At a 600-μm light guide thickness, the radiant flux was 20.58 mW, a 1.521-fold increase. Comparing the difference between simulation and measurement shows that the results are similar to those in the actual sample test.

**Table 4:** Difference between simulated and measured LEE of UV-C LEDs with light guide layer thicknesses of 150–700 μm

*H*_LG_ (µm)	Simulation results		Measured results	
	Relative radiant Flux (mW)	Radiant flux magnification	Relative radiant flux (mW)	Radiant flux magnification
150	0.41	1	13.53	1
300	0.429	1.046	14.1	1.042
350	0.5	1.219	16.49	1.218
400	0.536	1.307	17.76	1.312
500	0.594	1.448	19.74	1.458
600	0.62	1.512	20.58	1.521
700	0.634	1.546	20.86	1.541

## Conclusions

This paper proposes a first-order optical design using Al_2_O_3_ material as the light guide layer to reduce the absorption caused by TIR and optimize the LEE of UV-C LEDs. The effects of light guide layers of different thicknesses on the LEE of UV-C LEDs were simulated and analyzed using SPEOS optical simulation software. Compared with the standard layer thickness of 150 μm, an optimized thickness of 600 μm resulted in a 1.52-fold increase in LEE. This improved UV-C LED LEE is beneficial for the use of such LEDs in sterilization systems and other future applications.

## Data Availability

The datasets supporting the conclusions of this article are available in the article.

## Abbreviations

DUV: Deep ultraviolet
H_pd_: Electrode thickness
LEE: Light extraction efficiency
L: Length
LGL: Light guide layer
LE: Light emitting layer
TIR: Total internal reflection
UV-C LEDs: Ultraviolet-C light-emitting diodes
W: Width

## References

[1] Hirayama H, Maeda N, Fujikawa S, Toyoda S, Kamata N (2014). Recent progress and future prospects of AlGaN-based high-efficiency deep-ultraviolet light-emitting diodes. Jpn J Appl Phys.

[2] Kneissl M, Kolbe T, Chua C, Kueller V, Lobo N, Stellmach J, Knauer A, Rodriguez H, Einfeldt S, Yang Z, Johnson NM, MWeyers (2010). Advances in group III-nitride-based deep UV light-emitting diode technology. Semicond Sci Technol.

[3] Murashita S, Kawamura S, Koseki S (2017). Inactivation of Nonpathogenic Escherichia coli, Escherichia coli O157:H7, Salmonella enterica Typhimurium, and Listeria monocytogenes in Ice Using a UVC Light-Emitting Diode. J Food Prot.

[4] Shatalov M, Sun W, Lunev A, Xuhong Hu, Dobrinsky A, Bilenko Y, Yang J, Shur M, Gaska R, Moe C, Garrett G, Wraback M (2012). AlGaN deep-ultraviolet light-emitting diodes with external quantum efficiency above 10%. Appl Phys Express.

[5] Selma MV, Allende A, López-Galvez F, Conesa MA, Gil MI (2008). Disinfection potential of ozone, ultraviolet-C and their combination in wash water for the fresh-cut vegetable industry. Food Microbiol.

[6] Tremarin A, Brandao TRS, Silva CLM (2016). Inactivation kinetics of *Alicyclobacillus acidoterrestris* in apple juice submitted to ultraviolet radiation. Food Control.

[7] Kima DK, Kanga DH (2020). Inactivation efficacy of a sixteen UV-C LED module to control foodborne pathogens on selective media and sliced deli meat and spinach surfaces. LWT Food Sci Technol.

[8] Nyangaresi PO, Qin Y, Chen G, Zhang B, Lu Y, Shen L (2019). Comparison of the performance of pulsed and continuous UVC-LED irradiation in the inactivation of bacteria. Water Res.

[9] Kim DK, Kim SJ, Kang DH (2017). Bactericidal effect of 266 to 279 nm wavelength UVC-LEDs for inactivation of Gram positive and Gram negative foodborne pathogenic bacteria and yeasts. Food Res Int.

[10] Kuo SY, Chang CJ, Huang ZT, Lu TC (2020). Improvement of light extraction in deep ultraviolet GaN light emitting diodes with mesh P-contacts. Appl Sci.

[11] Pai YM, Lin CH, Lee CF, Lin CP, Huan C, Hao C, Kuo C, Ye ZT (2018). Enhancing the light-extraction efficiency of AlGaN-based deep- ultraviolet light-emitting diodes by optimizing the diameter and tilt of the aluminum sidewall. Curr Comput-Aided Drug Des.

[12] Inoue SI, Naoki T, Kinoshita T, Obata T, Yanagi H (2015). Light extraction enhancement of 265 nm deep-ultraviolet light-emitting diodes with over 90 mW output power via an AlN hybrid nanostructure. Appl Phys Lett. DOI.

[13] Sun W, Shatalov M, Deng J, Hu X, Yang J, Lunev A, Bilenko Y, Shur M, Gaska R (2010). Efficiency droop in 245–247 nm AlGaN light-emitting diodes with continuous wave 2 mW output power. Appl Phys Lett.

[14] Jain B, Velpula RT, Tumuna M, Bui HQT, Jude J, Pham TT, Van Le T, Viet Hoang A, Wang R, Nguyen HPT (2020). Enhancing the LEE of AlInN nanowire ultraviolet light-emitting diodes with photonic crystal structures. Opt Express.

[15] Lin R, Galan SV, Sun H, Hu Y, Alias MS, Janjua B, Ng TK, Ooi BS, Li X (2018). Enhancing the LEE of AlInN nanowire ultraviolet light-emitting diodes with photonic crystal structures. Photon Res.

[16] Ke WC, Lee F-W, Chiang C-Y, Liang Z-Y, Chen W-K, Seong T-Y (2016). InGaN-based light-emitting diodes grown on a micro/nanoscale hybrid patterned sapphire substrate. Appl Mater Interfaces.

[17] Manley P, Walde S, Hagedorn S, Hammerschmidt M, Burger S, Becker C (2020). Nanopatterned sapphire substrates in deep-UV LEDs: is there an optical benefit. Opt Express.

[18] Huang SH, Horng R-H, Wen K-S, Lin Y-F, Yen K-W, Wuu D-S (2006). Improved light extraction of nitride-based flip-chip light-emitting diodes via sapphire shaping and texturing. IEEE Photon Technol Lett.

[19] Kim DY, Park JH, Lee JW, Hwang S, Jae Oh S, Kim J, Sone C, Schubert EF, Kim JK (2015). Overcoming the fundamental light-extraction efficiency limitations of deep ultraviolet light-emitting diodes by utilizing transverse-magnetic-dominant emission.

[20] Liang R, Dai J, Xu L, He J, Wang S, Peng Y, Wang H, Ye L, Chen C (2018). High LEE of deep ultraviolet leds enhanced using nanolens arrays. IEEE Trans Electron Devices.

[21] Xie B, Hu R, Chen Q, Yu X, Wu D, Wang K, Luo X (2015). Design of a brightness-enhancement-film-adaptive freeform lens to enhance overall performance in direct-lit light-emitting diode backlighting. Appl Opt.

[22] Nagasawa Y, Hirano A (2019). Review of encapsulation materials for AlGaN-based deep-ultraviolet light-emitting diodes. Photon Res.

[23] Liu J, Liu J, Li S, Cheng H, Lei Z, Peng Y, Chen M (2021). Deep-ultraviolet LEDs with all-inorganic and hermetic packaging by 3D ceramic substrate. IEEE Photon Technol Lett.

[24] Yamada K, Furusawa Y, Nagai S, Hirano A, Ippommatsu M, Aosaki K, Morishima N, Amano H, Akasaki I (2014). evelopment of underfilling and encapsulation for deep-ultraviolet LEDs. Appl Phys Express.

[25] Kang CY, Lin CH, Wu T, Lee PT, ZC and HC Kuo,  (2019). A novel liquid packaging structure of deep-ultraviolet light-emitting diodes to enhance the light-extraction eciency. Curr Comput-Aided Drug Des.

[26] Lu CC, Wang CP, Liu CY, Hsu CP (2016). The efficiency and reliability improvement by utilizing quartz airtight packaging of UV-C LEDs. IEEE Trans Electron Dev.

[27] Ye ZT, Pai Y-M, Lin CH, Chen LC, Nguyen HT, Wang H-C (2019). Nanoparticle-doped polydimethylsiloxane fluid enhances the optical performance of AlGaN-based deep-ultraviolet light- emitting diodes. Nanoscale Res Lett.

[28] Peng Y, Guo X, Liang R, Cheng H, Chen M (2017). Enhanced light extraction from DUV-LEDs by AlN-doped fluoropolymer encapsulation. IEEE Photon Technol Lett.

[29] Yun J, Hirayama H (2021). Investigation of light-extraction efficiency of flip-chip AlGaN-based deep-ultraviolet light-emitting diodes adopting AlGaN metasurface. IEEE Phots J.

[30] Yang W, Chen J, Zhang Y, Zhang Y, He J-H, Fang X (2019). Silicon-compatible photodetectors: trends to monolithically integrate photosensors with chip technology. Adv Function Mater.

[31] Chen J, Ouyang W, Yang W, He J-H, Fang X (2020). Recent progress of heterojunction ultraviolet photodetectors: materials, integrations, and applications. Adv Function Mater.

[32] Guan X, Yu X, Periyanagounder D, Benzigar MR, Huang J-K, Lin C-H, Kim J, Singh S, Hu L, Liu G, Li D, He J-H, Yan F, Wang QJ, Wu T (2020). Recent progress in short- to long-wave infrared photodetection using 2D materials and heterostructures. ADVANCED OPTICAL MATERIALS.

[33] Manikandan A, Chen Y-Z, Shen C-C, Sher C-W, Kuo H-C, Chueh Y-L (2019). A critical review on two-dimensional quantum dots (2D QDs): From synthesis toward applications in energy and optoelectronics. Prog Quantum Electron.

[34] Wang H-P, Periyanagounder D, Li A-C, He J-H (2018). Fabrication of silicon hierarchical structures for solar cell applications. IEEE Access.

[35] Fu H-C, Ramalingam V, Kim H, Lin C-H, Fang X, Alshareef HN, He J-H (2019). MXene-contacted silicon solar cells with 11.5% efficiency. Adv Energy Mater.

[36] Liu Z, Lin C-H, Hyun B-R, Sher C-W, Lv Z, Luo B, Jiang F, Wu T, Ho C-H, Kuo H-C, He J-H (2020). Micro-light-emitting diodes with quantum dots in display technology. Light-Sci Appl.

